# Global prevalence and public health impact of intestinal parasitic infections in children under five: a systematic review and meta-analysis

**DOI:** 10.1186/s12889-026-27787-2

**Published:** 2026-05-18

**Authors:** Hassan Taherahmadi, Meysam Olfatifar, Sayed Hussain Mosawi, Morteza Habibi, Abouzar Babaei, Ioannis Adamopoulos, Fatemeh Samiee-Rad, Zahra Gharibi, Faezeh Mohammadi, Kareem Hatam-Nahavandi, Aida Vafae Eslahi, Milad Badri

**Affiliations:** 1 Children Growth Research Center, Research Institute for Prevention of Non-Communicable Diseases, Qazvin University of Medical Sciences, Qazvin, Iran; 2 Gastroenterology and Hepatology Diseases Research Center, Qom University of Medical Sciences, Qom, Iran; 3 Medical Sciences Research Center, Ghalib University, Kabul, Afghanistan; 4 Medical Microbiology Research Center, Qazvin University of Medical Sciences, Qazvin, Iran; 5 Department of Public Health Policy, Sector of Occupational and Environmental Health, School of Public Health, University of West Attica, Athens, Greece; 6 Department of Pathobiology, Faculty of Medical School, Qazvin University of Medical Sciences, Qazvin, Iran; 7 Kowsar Medical and Educational Center, Qazvin, Iran; 8 Infectious and Tropical Diseases Research Center, Hormozgan Health Institute, Hormozgan University of Medical Sciences, Bandar Abbas, Iran; 9 Department of Medical Parasitology and Mycology School of Medicine Qazvin, University of Medical Sciences, Qazvin, Iran; 10 Department of Parasitology and Mycology, School of Medicine, Iranshahr University of Medical Sciences, Iranshahr, Iran

**Keywords:** Intestinal parasitic infections, Children under five, Prevalence, Child health, Soil-transmitted helminths, Neglected tropical diseases, WASH

## Abstract

**Background:**

Intestinal parasitic infections (IPIs) remain a significant public health concern worldwide, particularly affecting children under five years old. These infections contribute to malnutrition, impaired growth, diarrhea, and increased morbidity and mortality, disproportionately impacting low- and middle-income countries. This systematic review and meta-analysis aims to estimate the global prevalence of IPIs in children under five and examine the influence of socioeconomic, environmental, and diagnostic factors.

**Methods:**

A systematic literature search was conducted based on Preferred Reporting Items for Systematic Reviews and Meta-Analyses (PRISMA) guidelines across multiple databases (PubMed, Web of Science, Scopus, ScienceDirect, and Google Scholar) for cross-sectional studies reporting the prevalence of IPIs in children under five years old, up to November 2025. The pooled prevalence was estimated using a random-effects meta-analysis, and subgroup analyses were performed by region, human development index (HDI), income level, climate, diagnostic method, and parasite species. Methodological quality was assessed using an adapted Newcastle–Ottawa Scale.

**Results:**

Forty-one studies, involving a total of 15,109 children under five, met the inclusion criteria. The study population was drawn from multiple regions worldwide. The overall pooled prevalence of IPIs in children under five was 31.60% (95% CI: 26.04–37.44). Prevalence was highest in lower-middle-income countries (37.45%) and low-HDI regions (35.17%), and lowest in high-income countries (6.25%) and very high-HDI regions (19.41%). South Asia (35.47%) and Eastern sub-Saharan Africa (35.24%) were identified as major hotspots. Helminth infections were most commonly caused by *Ascaris lumbricoides* (11.93%), while *Giardia lamblia* (10.37%) was the predominant protozoan. Studies using more sensitive diagnostic methods reported substantially higher prevalence. Environmental factors such as tropical savanna climates, moderate rainfall, and high humidity were associated with increased infection rates. Gender differences in prevalence were negligible.

**Conclusions:**

IPIs continue to pose a substantial global health burden on children under five, with socioeconomic and environmental disparities strongly influencing prevalence. The findings highlight the need for targeted public health interventions, including deworming programs, improved sanitation, and clinical surveillance, particularly in high-burden regions. Enhanced diagnostic strategies are essential to accurately capture infection rates and guide effective prevention and treatment efforts.

**Supplementary Information:**

The online version contains supplementary material available at 10.1186/s12889-026-27787-2.

## Background

Intestinal parasitic infections (IPIs) continue to pose a major threat to public health across the world [1]. These parasites are a major cause of global morbidity and mortality in endemic regions [2]. Intestinal parasites are commonly divided into protozoa and helminths. The data from World Health Organization (WHO) indicate that the soil-transmitted helminths (STHs), including *Ascaris lumbricoides*, hookworms, and *Trichuris trichiura* are among the most common parasitic infections globally [3, 4]. Beyond helminth infections, enteric protozoan infections remain a significant public health concern, largely due to their role in causing diarrheal illness in young children. Although diarrhea can result from various pathogens, *Entamoeba histolytica*, *Giardia duodenalis*, and *Cryptosporidium* species have been consistently identified as leading causative agents in pediatric populations worldwide [2, 5].

Children with poor nutritional status are highly vulnerable to IPIs, while persistent parasitic infections can, in turn, aggravate undernutrition, causing a lethal cycle of worsening illness and malnutrition [6, 7]. Together, these factors contribute substantially to high under-five mortality in developing and highly populated nations, with undernutrition accounting for almost 50% of such deaths [6]. In 2020, approximately 45.4 million children under five were affected by wasting, while 149.2 million experienced stunting worldwide. The Southeast Asia region was the most severely impacted, with over 30% of children affected by stunting and more than 15% by wasting during the same year [6, 8]. Although IPIs remain more prevalent in developing countries, incidence is rising in developed nations due to globalization as well, driven by globalization of the food supply, international travel, and migration [9].

In developed countries, migration from less-developed regions can contribute to public health challenges. Therefore, understanding its global prevalence helps facilitate the implementation of tertiary prevention measures, including the diagnosis and treatment of affected individuals [10, 11].

This systematic review and meta-analysis aims to provide a comprehensive assessment of the global prevalence of IPIs in children under five years of age. While numerous studies have examined IPIs at local or national levels, especially in endemic regions, there is limited synthesis of data across countries and continents [12–14]. The novelty of this study stems from its wide global coverage, its emphasis on the highly vulnerable population of children under five years old, and its incorporation of socioeconomic, environmental, and diagnostic factors that affect prevalence. In contrast to earlier research, this study explores geographic hotspots, climate conditions, income levels, and human development indices, providing a comprehensive and multidimensional perspective on the global disease burden.

## Methods

### Search strategy

**Fig. 1:**
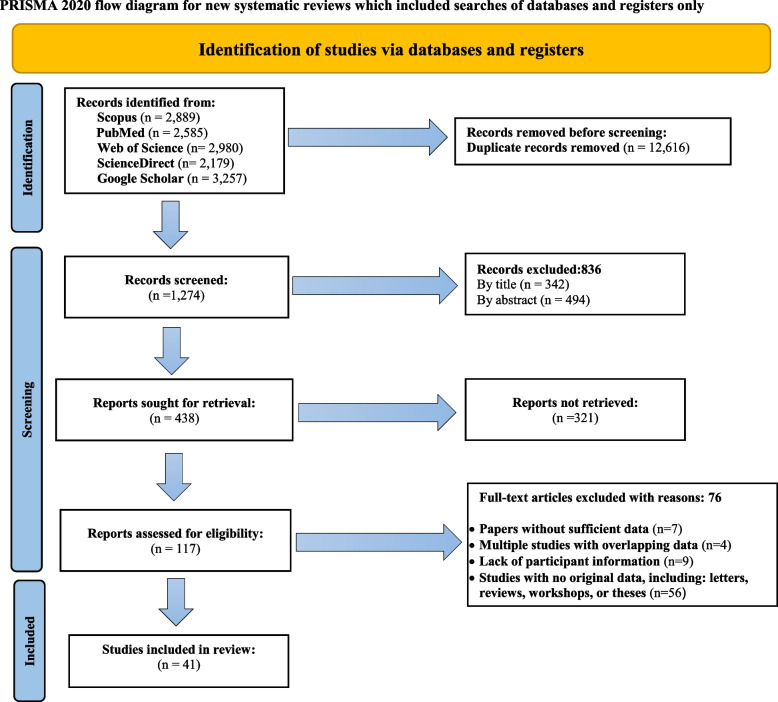
Flow diagram of the study design process

This systematic review and meta-analysis was conducted following the Preferred Reporting Items for Systematic Reviews and Meta-Analyses (PRISMA) guidelines [15] (Fig. 1 and Supplementary Table 1). A comprehensive search was performed across multiple databases, including PubMed, Web of Science, Scopus, ScienceDirect, and Google Scholar, to identify studies reporting intestinal parasitic infections in children under five years old.

Search terms, used individually or in combination with Boolean operators (AND/OR), included: epidemiology, prevalence, incidence, parasites, parasitic diseases, parasitic infections, helminthiasis, helminth parasites, helminthic infections, protozoan infections, protozoan pathogens, intestinal protozoans, preschool-age children, under five years old, young child, infant, global, and worldwide.

Additionally, the search strategy was designed to include literature from the following 19 countries: Bangladesh, Burkina Faso, Cambodia, Egypt, Ethiopia, India, Iran, Iraq, Kenya, Malaysia, Mexico, Mozambique, Nepal, Nigeria, Pakistan, Peru, the Philippines, Rwanda, and Saudi Arabia. After initial screening of titles and abstracts, duplicates and irrelevant studies were removed. The remaining full-text articles were independently reviewed by two authors (Z.G. and F.M.), and reference lists were examined to identify any additional eligible studies not captured in the database search.

### Inclusion and exclusion criteria

The following predefined inclusion criteria were applied: (1) peer-reviewed articles presenting original data, (2) published in English before November 2025, (3) cross-sectional studies reporting the prevalence of intestinal parasitic infections in children under five years old in any region of the world, (4) availability of both abstract and full text, and (5) reporting of numerator and denominator data sufficient to calculate prevalence. Studies that did not meet these criteria such as those in other languages, review articles without original data, letters, editorials, or articles with unclear or indeterminate results were excluded.

Data extraction was performed independently by two reviewers (Z.G. and F.M.) using a standardized Excel template, and a third reviewer (A.V.E.) cross-verified the entries to ensure accuracy. Extracted information included: first author, country and specific study location (province/city/district), year of publication, sample size, number of positive cases, participant gender, clinical manifestations, WHO region (African, Americas, South-East Asia, Eastern Mediterranean, Western Pacific), Global Burden of Disease (GBD) region (Andean Latin America, Central Latin America, Eastern sub-Saharan Africa, North Africa and Middle East, South Asia, Southeast Asia, Western sub-Saharan Africa), Human Development Index (HDI) (https://hdr.undp.org/data-center/human-development-index#/indicies/HDI), countries income level (https://datahelpdesk.worldbank.org/knowledgebase/articles/906519-world-bank-country-and-lending-groups), types of sample, diagnostic method, and species of parasites.

In addition, climatic variables were recorded for each study region, including humidity (https://www.timeanddate.com/weather/iran/tehran/climate), annual rainfall (https://en.climate-data.org/), average temperature (https://en.climate-data.org/), and climate (https://www.britannica.com/science/Koppen-climate-classification) (Tables 1, 2 and 3).

**Table 1:** Main characteristics of the included studies reporting the prevalence of intestinal helminthic and protozoan parasites among children under 5 years old

Study No	**Author**	**Study Year**	**Country**	**District / City / province**	**Diagnostic method**	**Sample size**	**Infected**	**Species of parasites**	**Clinical manifestations**
1	Daniel Gebretsadik et al	2018	Ethiopia	Dessie	Direct smear & Concentration (Sedimentation) & staining	232	36	*Entamoeba histolytica, Hymenolepis nana, Giardia lamblia, Schistosoma* spp.*, Enterobius vermicularis*	----
2	Getamesay Mulatu et al	2015	Ethiopia	Hawassa	Direct smear & Concentration (Sedimentation) & staining	158	42	*Entamoeba histolytica, Giardia lamblia, Cryptosporidium* spp.*, Ascaris lumbricoides, Hymenolepis nana, Trichuris trichiura*	----
3	Catrin E. Moore et al	2015	Cambodia	Siem Reap	Direct smear & Concentration (Sedimentation) & staining	318	159	Hookworm*, Strongyloides stercoralis, Enterobius vermicularis, Hymenolepis nana, Giardia lamblia, Blastocystis hominis, Entamoeba histolytica, Cryptosporidium* spp.*, Cyclospora cayetanensis*	Diarrhea, Abdominal pain, Anemia, Malnutrition
4	Filipa Santana Ferreira et al	2019	Mozambique	Nampula	Direct smear & Concentration (Sedimentation) & staining	831	263	*Giardia lamblia, Strongyloides stercoralis, Cryptosporidium* spp.*, Ascaris lumbricoides,* Hookworm*, Trichuris trichiura, Entamoeba histolytica, Hymenolepis nana, Cystoisospora belli*	Diarrhea, Malnutrition
5	Evariste Hakizimana et al	2023	Rwanda	Nyamasheke	Direct smear & Concentration (Sedimentation)	1048	557	*Ascaris lumbricoides, Trichuris trichiura,* Hookworm*, Taenia* spp.*, Giardia lamblia, Entamoeba histolytica, Entamoeba coli*	----
6	Telanesh Zemene and Melashu Balew Shiferaw	2018	Ethiopia	Debre Birhan	Direct smear & Concentration (Sedimentation)	247	43	*Entamoeba histolytica, Giardia lamblia, Ascaris lumbricoides, Trichuris trichiura, Taenia* spp.	----
7	Arsène W Zongo et al	2019	Burkina Faso	Ouagadougou	Direct smear & staining	317	66	*Entamoeba histolytica, Entamoeba coli, Giardia lamblia, Trichomonas intestinalis, Hymenolepis nana*	abdominal pain, vomiting, fever,constipation, anorexia, nausea and diarrhea
8	Fatemeh Mesgarian et al	2017	Iran	Gonbad-e Kavus	Direct smear & Concentration (Sedimentation) & staining	932	248	*Enterobius vermicularis, Blastocystis hominis, Giardia lamblia, Endolimax nana, Dientamoeba fragilis, Entamoeba coli, Iodamoeba buetschlii, Chilomastix mesnili, Trichomonas hominis*	----
9	Hossain Haratipour et al	2016	Iran	Shahroud	Direct smear & Concentration (Sedimentation) & Scotch Tape Test	1850	649	*Giardia lamblia, Hymenolepis nana, Enterobius vermicularis, Entamoeba Coli, Entamoeba Hartmanni, Dientamoeba Fragilis, Endolimax Nana, Iodamoeba Butschili, Blastocystis Homonis, Chilomastix Mesnili*	Malnutrition
10	Tadesse Duguma et al	2023	Ethiopia	Bachuma	Direct smear	323	95	*Ascaris lumbricoides, Trichuris trichiura, Giardia lamblia, Schistosoma.spp, Hymenolepis nana, Entamoeba histolytica,* Hook worm	----
11	Greisi Curico et al	2022	Peru	Iquitos	Direct smear & Concentration (Sedimentation) & PCR	144	16	*Strongyloides stercoralis, Trichuris trichiura, Ascaris lumbricoides,* Hook worm*, Ascaris lumbricoides*	----
12	Kristen Aiemjoy et al	2017	Ethiopia	Amhara	Direct smear & Concentration (Sedimentation)	212	138	*Ascaris lumbricoides, Trichuris trichiura, Giardia lamblia, Entamoeba histolytica, Entamoeba coli, Blastocystis hominis, Iodamoeba butschlii, Endolimax nana, Entamoeba hartmanni*	Diarrhea, Stunting, Underweight
13	Javier Gutiérrez-Jiménez et al	2018	Mexico	Chiapas	Direct smear & Concentration (Sedimentation)	178	60	*Trichuris trichiura, Hymenolepis nana, Enterobius vermicularis, Entamoeba histolytica, Giardia lamblia*	malnutrition, Underweight, Stunted
14	Adham Mohammad Hegazy et al	2014	Egypt	Damanhur	Direct smear & staining & kato-katz method	500	259	*Entamoeba histolytica, Giardia lamblia, Ascaris lumbricoides, Enterobius vermicularis,* Hook worm*, Hymenolepis nana*	Abdominal Colic, Constipation, Diarrhea, Vomiting, Fatigue, Peri anal itching, Pallor
15	Amanuel Yosep and Hunachew Beyene	2020	Ethiopia	Boricha Woreda	Direct smear & staining & kato-katz method	622	303	*Entamoeba histolytica, Giardia lamblia, Ascaris lumbricoides, Trichuris trichiura,* Hook worm*, Taenia* spp.*, Strongyloides stercoralis*	----
16	Muhammad Faisal Afridi et al	2020	Pakistan	Skardu	Direct smear & staining	300	161	*Ascaris Lumbricoides, Hymenolepis Nana, Cryptosporidium* spp.*, Giardia lamblia*	----
17	S. Awasthi and V.K. Pande	1997	India	Lucknow	Direct smear & staining	1061	185	*Ascaris lumbricoides, Giardia lamblia*	Malnutrition, low hemoglobin levels
18	Ralf Ignatius et al	2012	Rwanda	Butare	Direct smear & Concentration (Sedimentation) & PCR	582	233	*Ascaris lumbricoides, Giardia lamblia, Cryptosporidium* spp.*,* Hook worm*, Entamoeba histolytica, Trichuris trichiura, Strongyloides stercoralis, Balantidium coli, Entamoeba coli, Blastocystis hominis, Iodamoeba buetschlii, Trichomonas hominis, Endolimax nana, Chilomastix mesnili*	Abdominal distension
19	SM Sadjjadi and N Tanideh	2005	Iran	Marvdasht	Direct smear & Concentration (Sedimentation)	337	115	*Giardia lamblia, Entamoeba histolytica, Blastocystis hominis, Hymenolepis nana, Chilomastix mesnili, Ascaris lumbricoides, Trichuris trichiura, Iodamoeba buetschlii, Enterobius vermicularis*	Stunting, underweight, wasting
20	Parminder S. Suchdev et al	2014	Kenya	Kibera	Direct smear & Elisa	205	120	*Ascaris lumbricoides, Trichuris trichiura, Malaria* spp.	Anemia, Low ferritin, Underweight
21	Zulkifli A et al	1999	Malaysia	Kelantan	Direct smear & staining	268	127	*Ascaris lumbricoides, Trichuris trichiura,* Hook worm	Stunting, underweight, wasting
22	O. O. Omitola et al	2016	Nigeria	Ogun	Direct smear & Concentration (Sedimentation)	97	50	*Ascaris lumbricoides,* Hook worm	Stunting, underweight, wasting, thinness
23	A. Desiree LaBeaud et al	2015	Kenya	Msambweni	Direct smear & Concentration (Sedimentation) & PCR	545	216	*Ascaris lumbricoides, Malaria, Filaria, Schistosoma* spp.	----
24	Md. Shabab Hossain et al	2019	Bangladesh	Dhaka	Direct smear & Elisa	240	134	*Ascaris lumbricoides, Trichuris trichiura, Enterobius vermicularis, Giardia lamblia, Cryptosporidium* spp.*, Iodamoeba buetschlii*	Anemia
25	Al-Daoody and Al-Bazzaz	2020	Iraq	Erbil	Direct smear & Concentration (Sedimentation) & Scotch Tape Test	237	65	*Enterobius vermicularis*	----
26	Nagwa S.M. Aly et al	2010	Saudi Arabia	Tabuk	Direct smear & Concentration (Sedimentation) & staining	64	4	*Entameoba histolytica, Giardia lamblia, Cryptosporidium* spp.*, Hymenolepis Nana, Ascaris lumbricoides, Entrobius vermicularis*	----
27	Dhiren Subba Limbu et al	2021	Nepal	Dharan	Direct smear & Concentration (Sedimentation)	116	9	*Entamoeba histolytica, Hymenolepis nana, Giardia lamblia,* Hookworm*, Entamoeba coli, Ascaris lumbricoides*	----
28	P Pradhan et al	2013	Nepal	Kathmandu	Direct smear	26	11	*Entamoeba histolytica, Hymenolepis nana, Giardia lamblia, Trichuris trichiura,* Hook worm	----
29	M.H Anvari Tafti et al	2014	Iran	Yazd	Direct smear & Concentration (Sedimentation)	180	18	*Giardia lamblia, Blastocystis hominis, Chilomastix mesnili, Entamoeba Coli, Dientamoeba fragilis*	underweight
30	Bong-Jin KIM et al	2003	Philippines	Roxas	Direct smear & Concentration (Sedimentation)	301	35	*Ascaris lumbricoides, Trichuris trichiura,* Hook worm*, Entamoeba Coli*	----
31	Sachita Dhital et al	2016	Nepal	Kathmandu	Direct smear & Concentration (Sedimentation) & staining	238	78	*Entameoba histolytica, Giardia lamblia, Ascaris lumbricoides, Hymenolepis nana, Trichuris trichiura, Schistosoma* spp.*, Blastocystis hominis, Cryptosporidium* spp.*, Entamoeba Coli*	----
32	Harith Saeed Jaeffer	2011	Iraq	Baghdad	Direct smear & Concentration (Sedimentation)	513	53	*Entameoba histolytica, Giardia lamblia*	Diarrhea
33	Upama KC et al	2019	Nepal	Kathmandu	Direct smear & Concentration (Sedimentation)	20	16	*Ascaris lumbricoides, Trichuris trichiura,* Hook worm*, Hymenolepis nana, Entameoba histolytica, Giardia lamblia, Blastocystis hominis, Entamoeba Coli, Entamoeba hartmanni, Endolimax nana*	----
34	Daniel Njenga et al	2022	Kenya	Nairobi	Direct smear & staining & kato-katz method	406	110	*Ascaris lumbricoides, Trichuris trichiura,* Hook worm*, Entameoba histolytica, Giardia lamblia, Entamoeba Coli*	----
35	Bhattachan B et al	2015	Nepal	Chitwan	Direct smear & Concentration (Sedimentation)	63	17	*Giardia lamblia, Blastocystis hominis, Entamoeba Coli, Entameoba histolytica, Endolimax nana, Taenia* spp.*, Hymenolepis nana, Trichuris trichiura,* Hook worm	----
36	Showkat Ahmad Wani et al	2010	India	Kashmir	Direct smear & Concentration (Sedimentation) & Scotch Tape Test	91	46	*Ascaris lumbricoides, Trichuris trichiura, Taenia* spp.*, Entrobius vermicularis*	----
37	Rashid MK et al	2011	India	Bareilly	Direct smear	53	11	*Ascaris lumbricoides, Giardia lamblia, Entameoba histolytica, Hymenolepis nana, Trichuris trichiura, Taenia* spp.	itching
38	Khadejeh Salahi et al	2019	Iran	Zanjan	Direct smear & Concentration (Sedimentation) & Scotch Tape Test	137	9	*Giardia lamblia, Blastocystis hominis, Entamoeba Coli, Endolimax nana, Taenia* spp.*, Entrobius vermicularis*	----
39	Amulya Dahal et al	2022	Nepal	Kirtipur	Direct smear	76	17	*Ascaris lumbricoides, Trichuris trichiura,* Hook worm*, Hymenolepis nana, Taenia* spp.*, Entrobius vermicularis, Giardia lamblia, Entameoba histolytica*	----
40	Rawaa Abdulkhaleq Hussein et al	2011	Iraq	Baghdad	Direct smear & Concentration (Sedimentation)	285	134	*Giardia lamblia, Entameoba histolytica, Entamoeba Coli, Endolimax nana, Iodamoeba buetschlii, Blastocystis hominis, Ascaris lumbricoides, Hymenolepis nana, Taenia* spp.*, Entrobius vermicularis*	Abdominal pain, Diarrhea, Fever, Vomiting, Perianal pruritus, Bloody diarrhea, Rectal prolaps
41	Degu Abate et al	2025	Ethiopia	Shinile	Direct smear & staining	756	115	*Cryptosporidium* spp.	Diarrhea

**Table 2:** Sub–group analysis based on HDI, income level, types of sample, diagnostic method, average temperature, annual rainfall, climate, GBD geographic regions, WHO region, countries, humidity, gender and district/city/province in included studies

Variables	**No studies**	**Sample size**	**Infected**	**Pooled prevalence** **(95% CI)**	**Heterogeneity**
					***I***^***2***^** τ**^**2**^ ***p*-** **value**
HDI					
Very High human development	3	476	147	19.41 (2.15–47.33)	97 6.78 *P <* .001
High human development	8	4415	1393	24.81 (14.54–36.76)	97 3.4 *P <* .001
Medium human development	17	4493	1381	34.60 (25.58–44.20)	97 3.94 *P <* .001
Low human development	13	5725	2102	35.17 (26.07–44.84)	98 3.2 *P <* .001
Income Level					
High income level	1	64	4	6.25 (2.94–15.38)	- - -
Upper middle income level	11	5061	1494	24.95 (16.43–34.58)	97 3.06 *P <* .001
Lower middle income level	18	4656	1634	37.45 (28.44–46.90)	97 3.97 *P <* .001
Low income level	11	5328	1891	32.23 (22.77–42.49)	98 3.14 *P <* .001
Types of sample					
Stool	35	12,321	4044	29.99 (24.04–36.29)	97 3.84 *P <* .001
Stool & Blood	6	2788	979	41.08 (27.72–55.14)	98 3.02 *P <* .001
Diagnostic method					
Direct smear	4	478	134	27.26 (18.99–36.37)	43 0.59 *P =* .15
Direct smear & staining	5	2702	654	29.68 (15.38–46.38)	98 3.81 *P <* .001
Direct smear & Concentration (Sedimentation)	13	3597	1245	32.10 (19.87–45.70)	98 6.29 *P <* .001
Direct smear & Concentration (Sedimentation) & staining	7	2773	830	26.36 (16.57–37.48)	94 2.46 *P <* .001
Direct smear & Concentration (Sedimentation) & Scotch Tape Test	4	2315	769	28.11 (11.34–48.79)	96 4.59 *P <* .001
Direct smear & Concentration (Sedimentation) & PCR	3	1271	465	29.30 (11.89–50.60)	96 3.65 *P <* .001
Direct smear & staining & Kato-katz method	3	1528	672	42.34 (27.45–57.93)	97 1.86 *P <* .001
Direct smear & ELISA	2	445	254	57.09 (52.34–61.77)	0 < .0001 *P =* .57
Average temperature					
10_20	11	3975	1187	26.84 (15.9–39.37)	94 4.66 *P <* .001
> 20	30	11,134	3836	33.37 (27.06–40)	97 3.55 *P <* .001
Annual rainfall					
< 400	10	5035	1554	23.94 (14.19–35.28)	97 3.9 *P <* .001
400–1000	15	6050	2285	36.65 (28.10–45.64)	97 3.16 *P <* .001
1001–1500	10	1084	524	33.60 (20.78–47.74)	95 4.87 *P <* .001
> 1500	6	2040	660	29.68 (16.77–44.47)	97 3.57 *P <* .001
Climate					
Desert climate	14	4627	1508	30.71 (21.25–41.07)	98 4.12 *P <* .001
Semi-desert climate	6	3753	1105	21.30 (12.21–32.09)	96 2.24 *P <* .001
Tropical monsoon climate	8	954	357	37.20 (22.14–53.60)	94 5.09 *P <* .001
Tropical rainforest climate	3	713	178	21.61 (4.41–46.80)	98 5.52 *P <* .001
Tropical savanna climate	6	3617	1499	41.38 (31.90–51.19)	96 1.46 *P <* .001
Tropical wet-dry climate	4	1445	376	35.24 (17.12–55.86)	98 4.24 *P <* .001
GBD geographic regions					
Andean Latin America	1	144	16	11.11 (7.07–17.37)	- - -
Central Latin America	1	178	60	33.71 (27.16–40.92)	- - -
Eastern sub-Saharan Africa	13	6167	2271	35.24 (26.41–44.61)	97 3.02 *P <* .001
North Africa and Middle East	10	5035	1554	23.94 (14.19–35.28)	97 3.9 *P <* .001
South Asia	11	2284	685	35.47 (23.18–48.78)	96 4.73 *P <* .001
Southeast Asia	3	887	321	34.80 (12.02–62.09)	98 5.84 *P <* .001
Western sub-Saharan Africa	2	414	116	35.09 (9.65–66.25)	96 4.99 *P <* .001
WHO region					
African	15	6581	2387	35.20 (27.03–43.82)	97 2.94 *P <* .001
Americas	2	322	76	21.38 (4.35–46.33)	95 3.53 *P <* .001
Eastern Mediterranean	11	5335	1715	26.39 (16.15–38.10)	97 4.38 *P <* .001
South-East Asia	10	1984	524	33.60 (20.78–47.74)	95 4.87 *P <* .001
Western Pacific	3	887	321	34.80 (12.02–62.09)	98 5.84 *P <* .001
Countries					
Bangladesh	1	240	134	5.58 (4.95–6.19)	- - -
Burkina Faso	1	317	66	20.82 (16.61–25.55)	- - -
Cambodia	1	318	159	50 (44.54–55.46)	- - -
Egypt	1	500	259	51.80 (47.43–56.14)	- - -
Ethiopia	7	2550	772	30.06 (17.26–44.66)	98 4.07 *P <* .001
India	3	1205	242	28.49 (11.14–49.90)	95 3.50 *P <* .001
Iran	5	3436	1039	21.34 (10.48–34.74)	96 2.82 *P <* .001
Iraq	3	1035	252	26.79 (8.80–50.07)	98 4.47 *P <* .001
Kenya	3	1156	446	41.34 (24.37–59.44)	96 2.49 *P <* .001
Malaysia	1	268	127	47.39 (41.39–53.40)	- - -
Mexico	1	178	60	33.71 (27.11–40.90)	- - -
Mozambique	1	831	263	31.65 (27.80–34.34)	- - -
Nepal	6	539	148	32.73 (14.72–53.72)	92 6.31 *P <* .001
Nigeria	1	97	50	51.55 (41.69–61.30)	- - -
Pakistan	1	300	161	53.76 (48–59.30)	- - -
Peru	1	144	16	11.11 (6.47–16.91)	- - -
Philippines	1	301	35	11.63 (8.32–15.61)	- - -
Rwanda	2	1630	790	46.64 (34.21–59.28)	96 0.80 *P <* .001
Saudi Arabia	1	64	4	6.25 (3.08–15.48)	- - -
Humidity					
< 40	7	5066	1829	28.04 (13.46–45.47)	98 3.77 *P <* .001
40–75	30	8953	2732	32.04 (25.09–39.41)	97 4.00 *P <* .001
> 75	4	1090	462	34.99 (8.83–67.40)	97 4.12 *P <* .001
Gender					
Male	17	8559	1602	33.10 (25.34–41.34)	95 2.98 *P <* .001
Female	17	4272	1551	33.19 (25.34–41.54)	94 3.05 *P <* .001
District/City/province					
Amhara	1	212	138	65.09 (58.46–71.18)	- - -
Bachuma	1	323	95	29.41 (24.63–34.55)	- - -
Baghdad	2	798	187	26.51 (1.41–67.03)	99 8.99 *P <* .001
Bareilly	1	53	11	20.75 (12.66–33.75)	- - -
Boricha Woreda	1	622	303	48.71 (44.79–52.64)	- - -
Butare	1	582	233	40.03 (36.11–44.06)	- - -
Chiapas	1	178	60	33.71 (27.28–40.97)	- - -
Chitwan	1	63	17	26.98 (18.07–39.19)	- - -
Damanhur	1	500	259	51.80 (47.42–56.16)	- - -
Debre Birhan	1	247	43	17.41 (13.08–22.55)	- - -
Dessie	1	232	36	15.52 (11.41–20.72)	- - -
Dhaka	1	240	134	55.83 (49.51–61.99)	- - -
Dharan	1	116	9	7.76 (4.43–14.32)	- - -
Erbil	1	237	65	27.43 (22.21–33.48)	- - -
Gonbad-e Kavus	1	932	248	26.61 (23.81–29.49)	- - -
Hawassa	1	158	42	26.58 (20.47–34.05)	- - -
Iquitos	1	144	16	11.11 (7.18–17.46)	- - -
Kashmir	1	91	46	50.55 (40.54–60.51)	- - -
Kathmandu	3	284	105	50.51 (23.21–77.65)	88 5.33 *P <* .001
Kelantan	1	268	127	47.39 (41.51–53.35)	- - -
Kibera	1	200	120	58.54 (51.69–65.05)	- - -
Kirtipur	1	76	17	22.37 (14.77–33.08)	- - -
Lucknow	1	1061	185	17.44 (14.77–19.40)	- - -
Marvdasht	1	337	115	34.12 (29.15–39.28)	- - -
Msambweni	1	545	216	39.63 (35.10–43.58)	- - -
Nairobi	1	406	110	27.09 (22.92–31.56)	- - -
Nampula	1	831	263	31.65 (28.53–34.86)	- - -
Nyamasheke	1	1048	557	53.15 (50.12–56.17)	- - -
Ogun	1	97	50	51.55 (41.80–61.15)	- - -
Ouagadougou	1	317	66	20.82 (15.23–24.53)	- - -
Roxas	1	301	35	11.63 (8.52–15.77)	- - -
Shahroud	1	1850	649	35.08 (32.68–37.09)	- - -
Shinile	1	756	115	15.21 (12.47–17.64)	- - -
Siem Reap	1	318	159	50 (44.45–55.55)	- - -
Skardu	1	300	161	53.67 (48.01–59.26)	- - -
Tabuk	1	64	4	6.25 (2.56–15.08)	- - -
Yazd	1	180	18	10 (6.51–15.32)	- - -
Zanjan	1	137	9	6.57 (3.76–12.24)	- - -
Total	41				

**Table 3:** Sub-group analysis based on type of helminthic and protozoan parasites

Type of parasite	**No studies**	**Sample size**	**Infected**	**Pooled prevalence** **(95% CI)**	**Heterogeneity**
					***I*** **2** **τ2 ** ***p*** **-value**
Protozoan					
*Giardia lamblia*	23	11,423	1236	10.37 (7.80–13.26)	95 1.06 *P <* .001
*Blastocystis hominis*	8	4696	169	3.95 (1.52–7.42)	96 1.09 *P <* .001
*Cyclospora cayetanensis*	1	318	9	2.83 (1.47–5.26)	- - -
*Chilomastix mesnili*	5	3881	27	0.88 (0.13–2.15)	89 0.29 *P <* .001
*Cryptosporidium* spp.	7	3116	239	6.25 (3.03–10.47)	94 0.95 *P <* .001
*Dientamoeba fragilis*	3	2962	21	0.84 (0–2.76)	94 0.36 *P <* .001
*Entamoeba coli*	11	6229	519	6.56 (2.16–13.03)	98 3.34 *P <* .001
*Entamoeba hartmanni*	2	2062	6	0.50 (0–2.34)	81 0.26 *P <* .001
*Entamoeba histolytica/dispar*	16	6576	467	5.99 (3.63–8.86)	95 1.13 *P <* .001
*Endolimax nana*	5	3861	118	4.55 (0.43–12.44)	96 2.65 *P <* .001
*Iodamoeba buetschlii*	7	4463	56	1.38 (0.24–3.30)	90 0.66 *P <* .001
*Balantidium coli*	1	582	1	0.17 (0–0.08)	- - -
Helminth					
*Ascaris lumbricoides*	20	8551	1112	11.93 (7.42–17.33)	97 2.93 *P <* .001
*Hymenolepis nana/diminuta*	10	5038	81	1.69 (0.58–3.29)	89 0.58 *P <* .001
Hookworm	12	5412	182	3.20 (1.59–5.33)	91 0.76 *P <* .001
*Strongyloides stercoralis*	4	2353	78	3.23 (0.67–7.51)	94 0.91 *P <* .001
*Schistosoma* spp.	2	868	28	3.21 (2.08–4.57)	0 0.01 *P =* .051
*Trichomonas hominis*	3	1831	31	1.76 (0.08–5.25)	94 0.68 *P <* .001
*Trichuris trichiura*	14	5825	288	4.52 (2.19–7.60)	94 1.42 *P <* .001
*Enterobius vermicularis*	9	4902	473	4.70 (0.99–10.80)	97 3.03 *P <* .001
*Taenia* spp.	4	2202	26	1.08 (0.60–1.68)	0 0.02 *P <* .001

### Data synthesis and statistical analysis

Various statistical methods were employed to comprehensively evaluate the global prevalence of intestinal parasitic infections in children under five years old. The overall pooled prevalence was calculated with a 95% confidence interval (CI). A random-effects model using the Freeman-Tukey double arcsine transformation was applied to estimate the pooled prevalence. Publication bias was assessed using Egger’s funnel plot, Begg’s rank correlation test, the Luis Furuya-Kanamori (LFK) index, and the Doi plot [14]. An LFK index beyond ± 2 was considered indicative of major asymmetry, values between ± 1 and ± 2 indicated minor asymmetry, and values within ± 1 were interpreted as symmetry, suggesting no evidence of publication bias.

Heterogeneity across the included studies was assessed using Cochrane’s Q test and quantified with the *I*^2^ statistic, with *I*^2^ values interpreted according to established thresholds: 0–25% indicated low heterogeneity, 25–50% moderate heterogeneity, and 50–75% high heterogeneity, in line with current methodological recommendations [16]. A *p*-value < 0.05 was considered statistically significant. All analyses were conducted using the meta and metasens packages in R (version 3.6.1) [17].

### Study quality assessment

A modified version of the Newcastle–Ottawa Scale was used to assign a quality score. This score evaluated the appropriateness of the study design, recruitment strategy, response rate, representativeness of the sample, objectivity and reliability of outcome assessment, provision of a power calculation, and suitability of the statistical analyses. Any discrepancies in scoring were resolved through consensus, resulting in a final agreed-upon rating for each study [18]. Studies were rated across three domains: selection (5 stars max), comparability (2 stars max), and outcome (3 stars max) Supplementary Table 2.

## Results

**Fig. 2:**
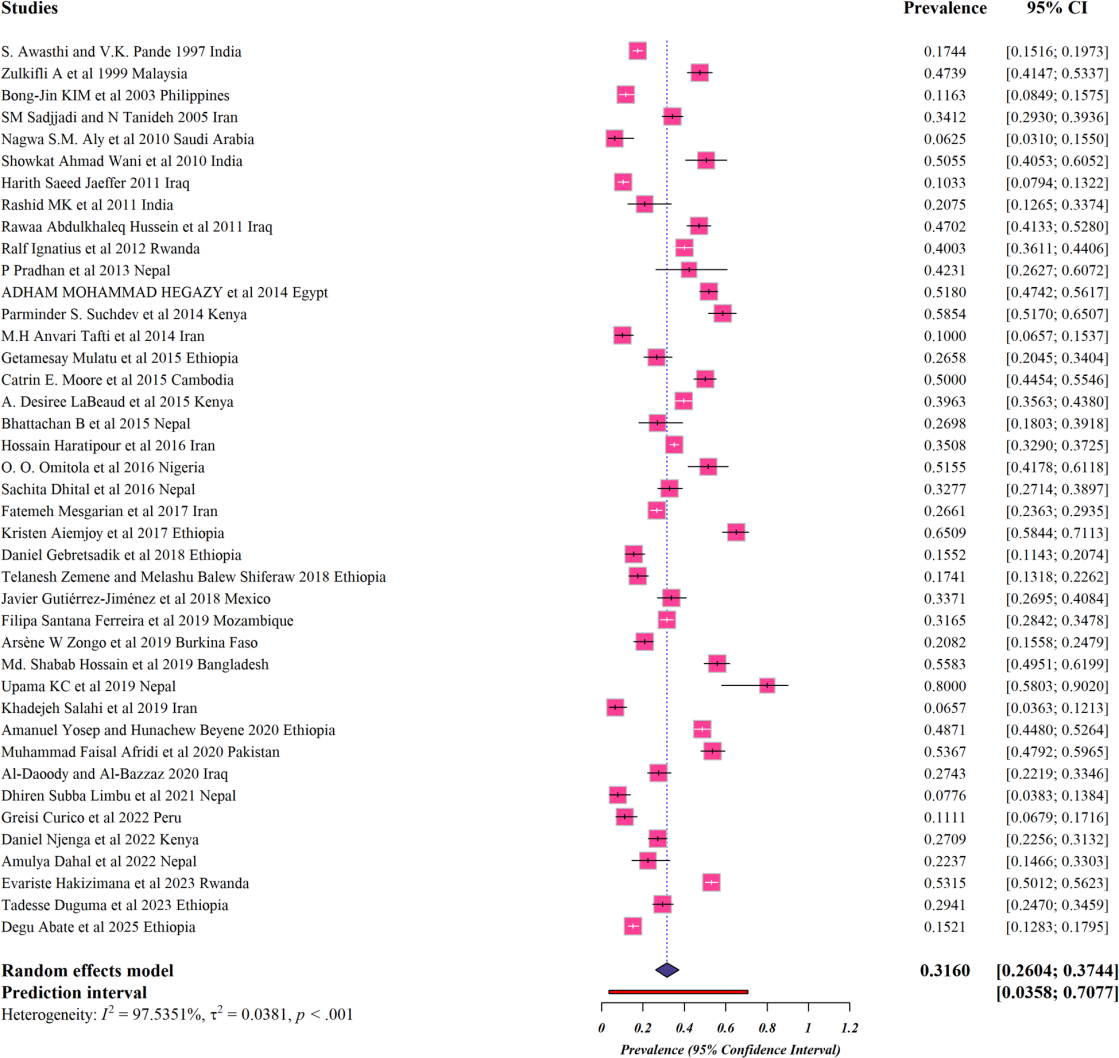
Forest plots for random-effects meta-analysis of intestinal parasitic infection in children under five years old (The boxes indicate the effect size of the studies (prevalence) and the whiskers indicate its confidence interval for corresponding effect size. There is no specific difference between white and black bars, only studies with a very narrow confidence interval are shown in white. In the case of diamonds, their size indicates the size of the effect, and their length indicates confidence intervals)

Based on the data from 41 studies included in this review indicates that the combined global prevalence of intestinal parasitic infections among children under five is 31.60% (95% CI: 26.04–37.44) (Fig. 2). The data highlight a pronounced socioeconomic divide, with the greatest burden observed in regions with constrained economic development. Children in lower-middle-income countries exhibited the highest prevalence at 37.45% (95% CI: 28.44–46.90), followed by those in low-income countries at 32.23% (95% CI: 22.77–42.49). In comparison, high-income countries showed a significantly reduced prevalence of 6.25% (95% CI: 2.94–15.38). Findings from HDI-based subgroup analyses reinforce this trend, demonstrating a strong negative correlation between a country's human development level and the prevalence of intestinal parasitic infections in young children.

The greatest burden of infection was found in countries with low HDI, where the prevalence reached 35.17% (95% CI: 26.07–44.84), followed closely by medium HDI nations at 34.60% (95% CI: 25.58–44.20). In contrast, prevalence declined steadily with higher levels of development. Countries classified as high HDI showed a substantially lower prevalence of 24.81% (95% CI: 14.54–36.76), and those with very high HDI reported the lowest rates at 19.41% (95% CI: 2.15–47.33).

The diagnostic approach used in the studies played a major role in determining the estimated prevalence of infection. Research employing more sensitive methods, specifically the combination of direct smear with enzyme-linked immunosorbent assay (ELISA) reported the highest pooled prevalence at 57.09% (95% CI: 52.34–61.77). Similarly, studies utilizing direct smear alongside staining techniques and the Kato-Katz method also identified a relatively high prevalence of 42.34% (95% CI: 27.45–57.93).

According to the GBD regional classification, the greatest prevalence of intestinal parasitic infections among children under five occurred in South Asia (35.47%, 95% CI: 23.18–48.78), based on 11 studies, and Eastern sub-Saharan Africa (35.24%, 95% CI: 26.41–44.61), based on 13 studies. In contrast, Andean Latin America reported the lowest prevalence at 11.11% (95% CI: 7.07–17.37), which included one study.

A similar trend was observed when using the broader WHO regional framework: The African Region (35.20%, 95% CI: 27.03–43.82), which includes Eastern sub-Saharan Africa, showed the highest infection burden, whereas the Region of the Americas (21.38%, 95% CI: 4.35–46.33) exhibited the lowest. These findings highlight significant geographic inequalities, with children in South Asia and Africa experiencing the highest risk of infection.

Country-level findings reveal pronounced disparities in the prevalence of intestinal parasitic infections. Extremely high rates were observed in Pakistan (53.76%, 95% CI: 48.01–59.30), Egypt (51.80%, 95% CI: 47.43–56.14), Nigeria (51.55%, 95% CI: 41.69–61.30), and Cambodia (50.0%, 95% CI: 44.54–55.46), indicating that roughly one in every two children under five is affected in these settings. Climatic conditions also played a critical role in shaping transmission patterns. The tropical savanna climate showed the highest infection prevalence at 41.38% (95% CI: 31.90–51.19), and regions with moderate annual rainfall (400–1000 mm) exhibited the most favorable conditions for parasite spread, with a prevalence of 36.65% (95% CI: 28.10–45.64). In contrast, semi-desert climates (21.30%, 95% CI: 12.21–32.09) and areas receiving low rainfall (< 400 mm) (23.94%, 95% CI: 14.19–35.28) had the lowest infection rates, highlighting the strong dependence of parasite survival and transmission on adequate moisture and temperature.

The subgroup analysis by humidity revealed a distinct environmental pattern: regions with high atmospheric moisture (> 75%) showed the greatest pooled prevalence of intestinal parasitic infections at 34.99% (95% CI: 8.83–67.40). This was followed by areas with moderate humidity (40–75%), where the prevalence reached 32.04% (95% CI: 25.09–39.41). In contrast, locations characterized by low humidity (< 40%) exhibited the lowest prevalence at 28.04% (95% CI: 13.46–45.47). These findings underscore that higher humidity levels serve as an important ecological factor contributing to increased transmission and infection rates.

Similarly, the subgroup analysis stratified by gender indicated minimal variation between males and females: the pooled prevalence was 33.10% (95% CI: 25.34–41.34) among males and 33.19% (95% CI: 25.34–41.54) among females, suggesting that infection burden is essentially comparable across sexes.

The district-, city-, and province-level analysis revealed substantial geographical heterogeneity in the prevalence of intestinal parasitic infections, identifying distinct high-burden areas. The most severe prevalence was observed in Amhara, Ethiopia, where infection rates reached 65.09% (95% CI: 58.46–71.18), indicating that nearly two-thirds of children were affected. This was followed by notably high rates in Kibera, Kenya (58.54%, 95% CI: 51.69–65.05) and Skardu, Pakistan (53.67%, 95% CI: 48.01–59.26). Conversely, the lowest prevalence estimates were recorded in Tabuk, Saudi Arabia (6.25%, 95% CI: 2.56–15.08), with similarly low levels in Zanjan, Iran (6.57%, 95% CI: 3.76–12.24), and Dharan, Nepal (7.76%, 95% CI: 4.43–14.32). The species-specific analysis revealed notable differences in the distribution of intestinal parasites. Among helminths, *A. lumbricoides* exhibited the highest prevalence at 11.93% (95% CI: 7.42–17.33), whereas *Taenia* spp. showed the lowest prevalence at 1.08% (95% CI: 0.60–1.68). In terms of protozoan infections, *G. lamblia* emerged as the most prevalent species with a pooled estimate of 10.37% (95% CI: 7.80–13.26), while *Balantidium coli* had the lowest prevalence at 0.17% (95% CI: 0.00–0.08).

**Fig. 3:**
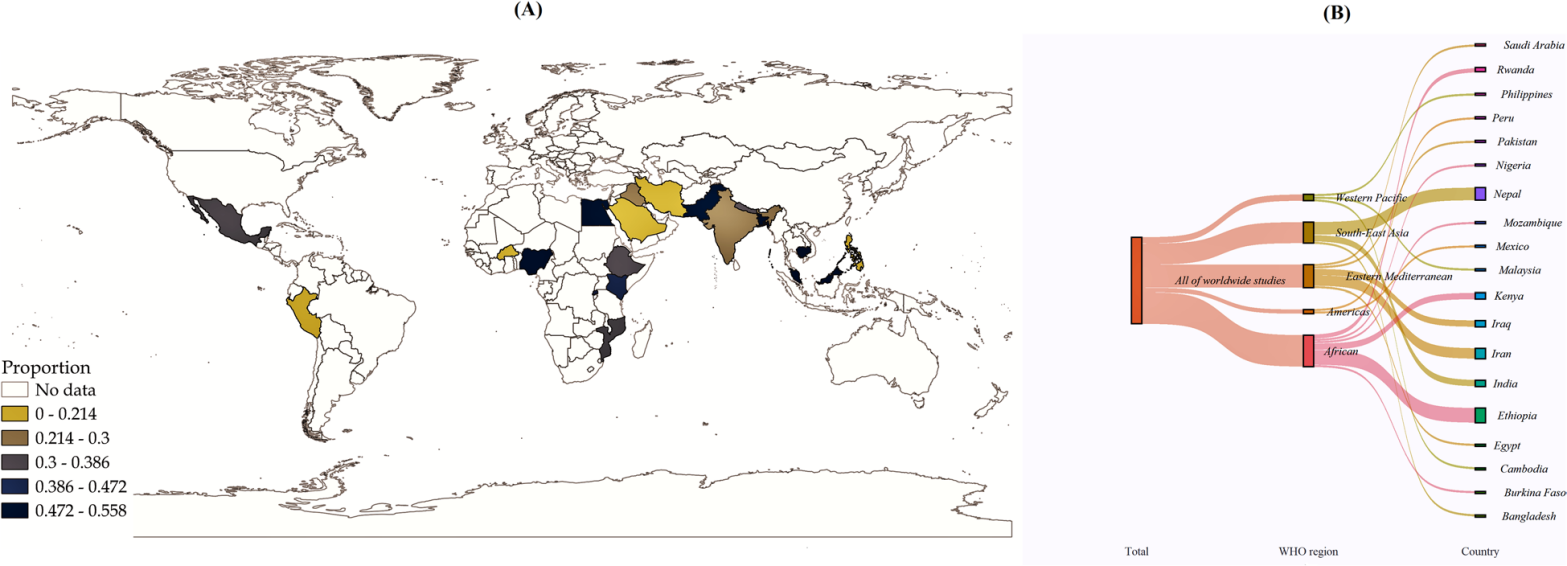
The global prevalence of intestinal parasitic infection in children under five years old in different geographical regions of the world based on included studies (https://qgis.org/en/site/) (**A**). Additionally, the Sankey plot presents data concerning the majority of studies related to countries and WHO Region based on the included studies (**B**)

Based on included studies the global distribution of intestinal parasites in children under five was visualized using QGIS3 software (https://qgis.org/en/site/) (Fig. 3A). Furthermore, the analysis of study distribution using a Sankey diagram (Fig. 3B), the African region was identified as the most prevalent WHO region, with Ethiopia being the most studies country.

### Meta regression

**Fig. 4:**
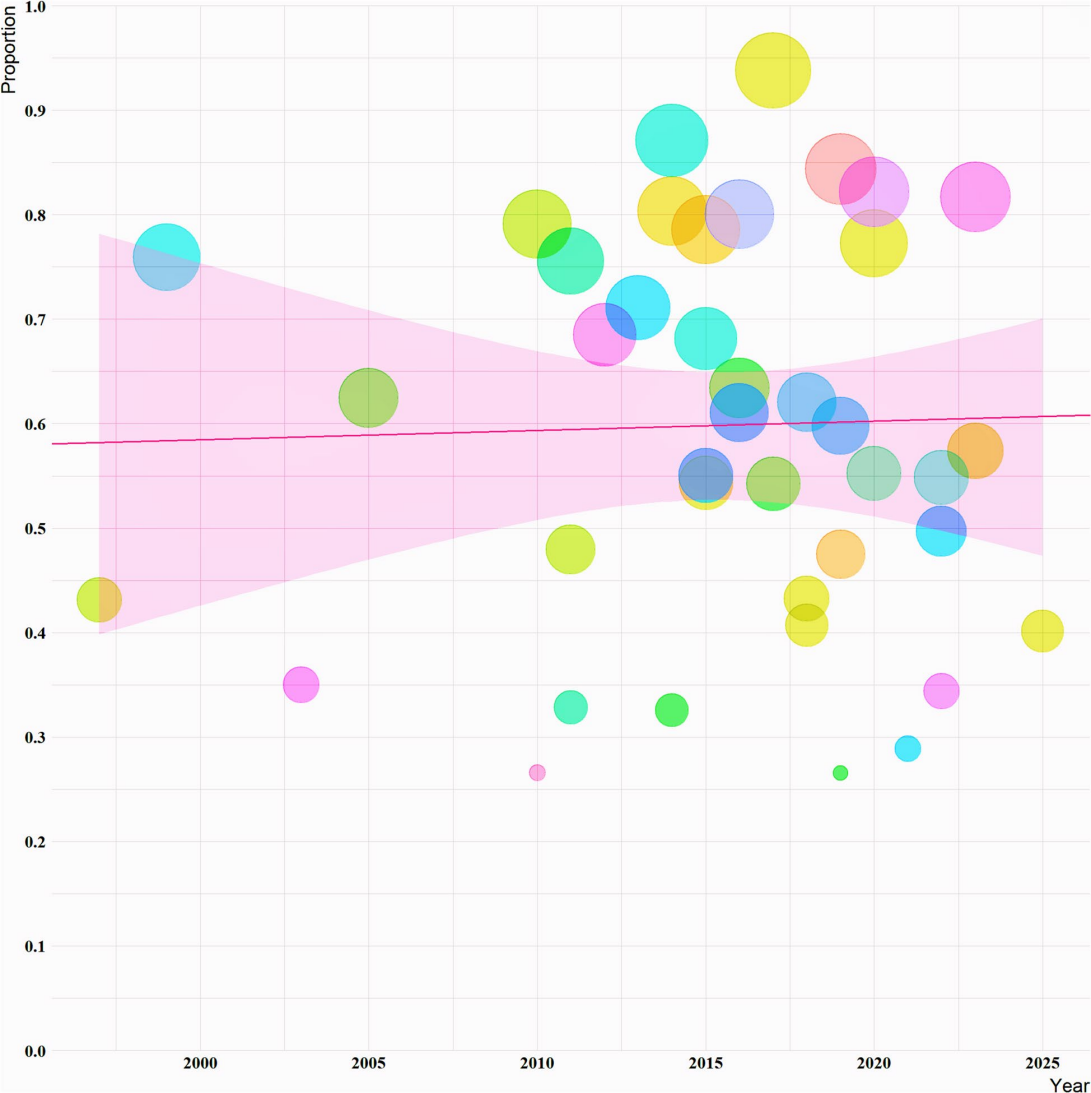
The global prevalence of intestinal parasitic infections among children under five years old in different geographical regions of the world based on year of publication (the pink line is the regression line, which was plotted based on the intercept and the slope of the regression model). The different coloured bubbles represent the countries under study, and their sizes indicate the effect size of each study

Meta-regression showed no significant association between year of publication and effect size (slope = 1.207, *P =* 0.860), indicating that publication year did not explain the between-study heterogeneity (Fig. 4).

### Publication bias

**Fig. 5:**
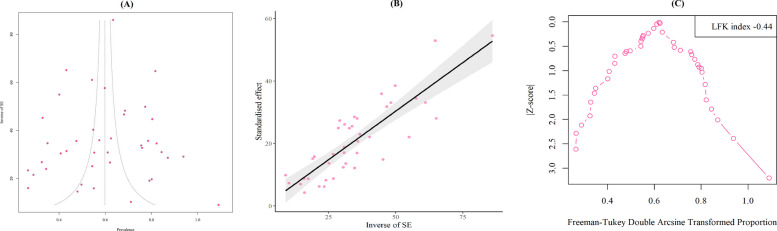
To evaluate potential publication bias in the estimated global prevalence of intestinal parasitic infections among children under five years old, Egger's funnel plot (**A**) and Begg's plot (**B**) were generated. In these plots, which use colored circles to represent individual studies, the effect size is marked by a central line, flanked by its confidence intervals. Furthermore, the Doi plot (**C**) provided a quantitative measure, with a Luis Furuya-Kanamori (LFK) index of 0.44, confirming the absence of significant asymmetry

There was no evidence of publication bias. Egger’s test (Z = 0.49, *P =* 0.621) and Begg’s test (t = 0.31, *P =* 0.761) were non-significant, and the Doi plot demonstrated no notable asymmetry (LFK index = 0.44), indicating the absence of small-study effects (Fig. 5 A-C).

### Quality assessment

Based on the quality assessment, 24 studies were high quality (scores 7–9) and 17 were of moderate quality (scores 4–6) (Supplementary Table 2).

## Discussion

This systematic review and meta-analysis provides an important and timely overview of the global impact of IPIs among children under five years of age. The estimated pooled prevalence underscores that IPIs continue to pose a widespread and serious public health concern, disproportionately affecting young children who are among the most vulnerable worldwide. The findings suggest that nearly one in three children in this age group is infected with at least one intestinal parasite, carrying significant consequences for early growth, cognitive development, and overall survival.

A key outcome of this review is the unmistakable socioeconomic divide in the distribution of IPIs. The highest burden falls on children in low- and lower-middle-income countries, whereas rates in high-income settings are substantially lower. This pattern is further supported by the strong negative association with the Human Development Index (HDI), with nations scoring lowest on the HDI experiencing the greatest prevalence. Such disparities are not random; they are fundamentally driven by unequal access to critical resources, including safe water, improved sanitation, suitable living conditions, and adequate healthcare systems [9, 19–21].

Poverty fosters environments in which fecal–oral transmission of intestinal parasites can easily occur. Moreover, these results clearly demonstrate the “vicious cycle” outlined earlier: IPIs lead to malnutrition through diarrhea, impaired nutrient absorption, and nutrient depletion, while malnourished children, whose immune defenses are weakened, become even more vulnerable to recurrent and severe parasitic infections [6, 7, 22]. Our results confirm that this cycle is a significant driver of childhood morbidity and a key obstacle to reducing under five mortality in developing regions.

Geographically, the findings identify South Asia and Eastern sub-Saharan Africa as having the highest burden of intestinal parasitic infections, which corresponds closely with worldwide patterns observed for other poverty-associated neglected tropical diseases and diarrheal conditions [8, 23]. The country-level analysis further highlights Pakistan, Egypt, Nigeria, and Cambodia as critical settings where approximately every second child under five is infected. This level of detail is crucial for directing international aid and national public health resources to where they are most urgently needed. Environmental drivers of transmission were clearly elucidated in our sub-group analyses. The tropical savanna climate, characterized by warm temperatures and seasonal rainfall, demonstrated the highest infection rate. Moderate annual rainfall (400–1000 mm) was the most conducive for transmission, as it provides the necessary moisture for egg and cyst maturation and survival in the soil without washing them away [9, 24, 25]. Correspondingly, high humidity (> 75%) was associated with a higher pooled prevalence. These conditions are ideal for the development and environmental survival of geohelminths like *A. lumbricoides* and *T. trichiura*, as well as for the prolonged viability of protozoan cysts [18, 24, 25]. Conversely, the significantly lower prevalence in semi-desert climates and areas with low annual rainfall (< 400 mm) underscores the environmental constraints on parasite survival outside the human host.

These findings carry distinct public health implications that shift away from universal mass drug administration (MDA) toward ecologically-informed intervention strategies. The identification of the 400–1000 mm rainfall belt as a high-transmission zone suggests that resources for MDA and sanitation infrastructure should be prioritized in these isohyetic zones, particularly in sub-Saharan Africa and parts of South America where tropical savannas predominate [26]. Furthermore, in high-transmission zones, water, sanitation, and hygiene (WASH) interventions must move beyond basic latrine construction to include drainage management. Reducing standing water and lowering household-level humidity through improved ventilation and flooring (e.g., replacing earthen floors with concrete) can break the environmental lifecycle of these parasites at the household unit [27].

A critical methodological insight from this review is the substantial influence of diagnostic sensitivity on reported prevalence estimates. Studies using a combination of Direct smear and ELISA demonstrated considerably higher prevalence rates compared to the overall pooled estimate, highlighting how diagnostic choice can affect observed burden. However, this finding should be interpreted cautiously, as it represents an inference derived from subgroup comparisons rather than a direct measurement of the true infection burden. Nevertheless, it provides indirect evidence suggesting that infections such as *G. lamblia* and *Cryptosporidium* spp. may be underdetected in field settings that rely primarily on less sensitive, single-method microscopy [5, 14, 28, 29]. The Kato-Katz method, while the gold standard for soil-transmitted helminths, misses protozoans entirely [30]. Therefore, our findings advocate for the use of more sensitive, multiplex diagnostic approaches in future epidemiological surveys to capture the true scale of the problem.

At the species level, *A. lumbricoides* and *G. lamblia* were identified as the dominant helminth and protozoan, respectively. This is consistent with global reports that highlight the ubiquity of these pathogens in settings with poor sanitation [31, 32]. The near-identical prevalence between males and females suggests similar exposure risks within household and community environments for this young age group, where behavioral differences are still limited. Public health interventions must therefore be tailored and implemented at a sub-national level to effectively target these high-risk settings.

### Limitations

Our findings are subject to certain limitations. First, the restriction to English-language publications may have introduced a selection bias, potentially omitting relevant data from non-English speaking regions. Second, the significant statistical heterogeneity observed, while common in global meta-analyses, indicates that there are residual factors influencing prevalence that our model could not capture. Third, the lack of uniformly reported, granular data on key confounders such as specific WASH practices, education, and detailed household income limited our ability to adjust for these critical variables in the analysis. Fourth, the wide confidence intervals observed for certain subgroups indicate substantial uncertainty, as these estimates are based on a limited number of studies; therefore, these data should be interpreted with caution. Fifth, The use of ecological climate data may not capture micro-environmental variations that modify true exposure. Despite these limitations, this study represents the most comprehensive and systematic assessment to date of the global prevalence of intestinal parasitic infections among children under five years old.

## Conclusions

This systematic review demonstrates that intestinal parasitic infections remain a significant and inequitable public health burden among children under five years of age, with the highest prevalence concentrated in low- and lower-middle-income countries, particularly in South Asia and Eastern sub-Saharan Africa, and with pronounced micro-geographical clustering in high-burden settings such as Amhara, Ethiopia. The findings further indicate that the observed burden is shaped by interacting socioeconomic and environmental determinants, including poverty, inadequate water and sanitation infrastructure, and climatic factors such as temperature and humidity, while also being likely underestimated due to the continued reliance on low-sensitivity diagnostic methods. To address these limitations, surveillance and national surveys should transition from conventional microscopy-based approaches to more sensitive diagnostic tools such as quantitative PCR, multiplex molecular assays, and enhanced concentration techniques, which enable improved detection of low-intensity infections and polyparasitism; these methods should be operationally embedded within routine sentinel surveillance systems and integrated into national health information platforms to improve spatial mapping of transmission and support evidence-based resource allocation. In parallel, effective control requires the implementation of hyper-localized, integrated public health interventions at district and sub-district levels, where mass drug administration is strategically combined with context-specific WASH interventions, including expansion of safe water access, school- and community-based sanitation infrastructure, and sustained hygiene behavior change programs, thereby enabling a more precise, efficient, and context-adapted response to persistent transmission hotspots.

## Supplementary Information

Supplementary Material 1.

Supplementary Material 2.

## Data Availability

The datasets generated and/or analyzed during this study are available from the corresponding author upon reasonable request.

## Abbreviations

IPI: Intestinal parasitic infection
GBD: Global Burden of Disease
WHO: World Health Organization
STHs: Soil-transmitted helminths
PRISMA: Preferred Reporting Items for Systematic Reviews and Meta-Analyses
HDI: Human Development Index
ELISA: Enzyme-linked immunosorbent assay
CI: Confidence intervals
WASH: Water, Sanitation, and Hygiene
