# Supplementary material for: Global prevalence and public health impact of intestinal parasitic infections in children under five: a systematic review and meta-analysis
Source: BMC Public Health. 2026 May 18;26:2095. doi: 10.1186/s12889-026-27787-2 (PMC13348570; doi:10.1186/s12889-026-27787-2)
Supplement: Supplementary file 2 — Supplementary Material 2. [file 12889_2026_27787_MOESM2_ESM.docx]

**Supplementary Table 2.** Quality assessment using the Newcastle–Ottawa scale modified for cross-sectional studies

| **No.** | **First author** | **Year** | **Selection**  **(maximum of 5 stars)** | **Comparability**  **(maximum of 2 stars)** | **Outcome**  **(maximum of 3 stars)** | **Total Score** |
| --- | --- | --- | --- | --- | --- | --- |
| 1 | Daniel Gebretsadik et al | 2018 | ** | * | ** | 5 |
| 2 | Getamesay Mulatu et al | 2015 | *** | * | *** | 7 |
| 3 | Catrin E. Moore et al | 2015 | *** | * | *** | 7 |
| 4 | Filipa Santana Ferreira et al | 2019 | *** | * | ** | 6 |
| 5 | Evariste Hakizimana et al | 2023 | ** | * | ** | 5 |
| 6 | Telanesh Zemene and Melashu Balew Shiferaw | 2018 | *** | ** | *** | 8 |
| 7 | Arsène W Zongo et al | 2019 | *** | * | ** | 6 |
| 8 | Fatemeh Mesgarian et al | 2017 | ** | * | ** | 5 |
| 9 | Hossain Haratipour et al | 2016 | *** | ** | *** | 8 |
| 10 | Tadesse Duguma et al | 2023 | *** | * | *** | 7 |
| 11 | Greisi Curico et al | 2022 | ** | * | ** | 5 |
| 12 | Kristen Aiemjoy et al | 2017 | *** | * | ** | 6 |
| 13 | Javier Gutiérrez-Jiménez et al | 2018 | *** | ** | *** | 8 |
| 14 | ADHAM MOHAMMAD HEGAZY et al | 2014 | ** | * | ** | 5 |
| 15 | Amanuel Yosep and Hunachew Beyene | 2020 | **** | ** | ** | 8 |
| 16 | Muhammad Faisal Afridi et al | 2020 | ** | * | ** | 5 |
| 17 | S. Awasthi and V.K. Pande | 1997 | *** | * | *** | 7 |
| 18 | Ralf Ignatius et al | 2012 | *** | ** | *** | 8 |
| 19 | SM Sadjjadi and N Tanideh | 2005 | ** | ** | ** | 6 |
| 20 | Parminder S. Suchdev et al | 2014 | *** | ** | *** | 8 |
| 21 | Zulkifli A et al | 1999 | *** | * | *** | 7 |
| 22 | O. O. Omitola et al | 2016 | *** | ** | *** | 8 |
| 23 | A. Desiree LaBeaud et al | 2015 | *** | * | *** | 7 |
| 24 | Md. Shabab Hossain et al | 2019 | ** | * | ** | 5 |
| 25 | Al-Daoody and Al-Bazzaz | 2020 | *** | * | ** | 6 |
| 26 | Nagwa S.M. Aly et al | 2010 | **** | ** | *** | 9 |
| 27 | Dhiren Subba Limbu et al | 2021 | *** | * | *** | 7 |
| 28 | P Pradhan et al | 2013 | ** | * | ** | 5 |
| 29 | M.H Anvari Tafti et al | 2014 | **** | ** | ** | 8 |
| 30 | Bong-Jin KIM et al | 2003 | **** | * | ** | 7 |
| 31 | Sachita Dhital et al | 2016 | ** | * | ** | 5 |
| 32 | Harith Saeed Jaeffer | 2011 | ** | * | ** | 5 |
| 33 | Upama KC et al | 2019 | *** | * | *** | 7 |
| 34 | Daniel Njenga et al | 2022 | *** | ** | ** | 7 |
| 35 | Bhattachan B et al | 2015 | **** | * | *** | 8 |
| 36 | Showkat Ahmad Wani et al | 2010 | ** | * | ** | 5 |
| 37 | Rashid MK et al | 2011 | *** | ** | *** | 8 |
| 38 | Khadejeh Salahi et al | 2019 | ** | ** | ** | 6 |
| 39 | Amulya Dahal et al | 2022 | *** | * | *** | 7 |
| 40 | Rawaa Abdulkhaleq Hussein et al | 2011 | *** | ** | *** | 8 |
| 41 | Degu Abate et al | 2025 | ** | ** | ** | 6 |

*Indicates one criteria was followed, ** two criteria were followed, ***three criteria were followed, ****four criteria were followed, and ***** five criteria were followed

**1) Representativeness of the Sample:**
a) Accurately reflects the general characteristics of the target population (e.g., entire population or random sampling).
b) Partially reflects the general characteristics of the target population (e.g., non-random sampling).
c) A specifically chosen or convenience group.
d) No information provided regarding the sampling method.

**2) Sample Size:**
a) Adequate and justified based on study design or rationale.
b) Not justified or insufficient rationale provided.

**3) Non-Respondents:**
a) Adequate response rate with demonstrated similarity between participants and non-participants in key characteristics.
b) Inadequate response rate or lack of comparability between participants and non-participants.
c) No information available regarding response rate or characteristics of respondents and non-respondents.

**4) Ascertainment of Exposure (Risk Factor):**
a) Use of a validated measurement instrument.
b) Use of a measurement instrument that is not validated, but its content or source is described or accessible.
c) No description of the instrument used for exposure measurement.

**Comparability (Maximum 2 stars):**

1. Study groups (e.g., compared outcomes) are comparable through study design or statistical adjustment, with control for confounding factors.
   a) Control for the most significant confounding factor (to be selected per study).
   b) Control for additional relevant confounding factors.

**Outcome (Maximum 3 stars):**

1. **Assessment of Outcome:**
   a) Outcome assessed by an independent evaluator who was blinded to exposure status.
   b) Outcome verified through record linkage (e.g., Available laboratory records for each patient).
   c) Outcome based on self-report.
   d) Method of outcome assessment not described.
2. **Statistical Test:**
   a) Statistical test is clearly described, appropriate for the data, and results are reported with measures of association (e.g., odds ratio, risk ratio) including confidence intervals and p-value.
   b) Statistical test is inappropriate, not clearly described, or incomplete in reporting.

This assessment scale is adapted from the Newcastle-Ottawa Quality Assessment Scale for cohort studies. It was modified to evaluate the methodological quality of cross-sectional studies included in the systematic review titled: Global Epidemiology and Public Health Impact of Intestinal Parasitic Infections in Children Under Five Years: A Systematic Review and Meta-Analysis.
